# FDA Accelerated Approval for Malignant Hematology and Oncology Indications in the Canadian Environment

**DOI:** 10.3390/curroncol29020036

**Published:** 2022-01-18

**Authors:** Cheryl Ho, Howard J. Lim, Dean A. Regier

**Affiliations:** 1 Department of Medical Oncology, BC Cancer, Vancouver, BC V5Z 4E6, Canada; hlim@bccancer.bc.ca; 2 Department of Medicine, University of British Columbia, Vancouver, BC V6T 1Z4, Canada; 3 Cancer Control Research, BC Cancer Research Institute, Vancouver, BC V5Z 1L3, Canada; dregier@bccrc.ca; 4 School of Population and Public Health, University of British Columbia, Vancouver, BC V6T 1Z4, Canada

**Keywords:** accelerated approval, FDA, Health Canada, health technology assessment, CADTH

## Abstract

Accelerated approval (AA) by the FDA enables earlier access to promising new therapies. Health Canada has a similar process. Canada implemented a national health technology assessment (HTA) for reimbursement decisions in 2011. This study evaluated regulatory and funding timelines and decisions for FDA AA cancer therapies in Canada. The FDA’s AA of malignant hematology and oncology from January 2000–December 2019 was reviewed. Dates from Health Canada, HTA decisions and provincial listings were collected. There were 94 FDA AAs, two of which were subsequently withdrawn. Of the 92 AAs, 70 received full (46)/conditional (24) Health Canada approval, and 22 were not filed. Since the introduction of HTA, 31 out of 45 of Health Canada’s approved indications underwent HTA review: 18 received a positive recommendation conditional on cost-effectiveness, 8 were not recommended and 5 were withdrawn/suspended. The median time from the AA to any Health Canada approval is 9.4 months, from any Health Canada approval to HTA decision is 5.8 months and from HTA decision to the first formulary listing is 12.0 months. The access and timeline for the first formulary listing differences were observed between the USA and Canada due to the decision of pharmaceutical companies to submit (or not) to regulatory/HTA bodies, national procedural delays with different healthcare delivery models and submission timelines. This study demonstrates that there is delayed access to promising new therapies in Canada.

## 1. Introduction

Regulatory approval is one of the critical steps in the complex process of drug development that facilitates access to new medications for diseases. In the United States (USA), the Food and Drug Administration (FDA) is responsible for the review of New Drug Applications, or Biologics License Applications, which may result in the authorization of the medication if sufficient evidence in support of the efficacy/effectiveness, safety, and quality in manufacturing is determined. In Canada, a similar review process is undertaken by Health Canada. The rigorous evaluation of submissions in support of new therapies can be lengthy, but this crucial process enables informed decision making regarding the risks and benefits of treatments.

In serious or life-threatening diseases such as cancer, there is tension between the standard approval process and clinicians’ and patients’ demand for early market access to promising and often expensive treatments. To address this issue in the USA, the FDA initiated expedited pathways, including the Accelerated Approval (AA) process [1]. AA enables the approval of therapies for serious illnesses on the basis of a surrogate or intermediate clinical endpoint or in light of non-randomized data while additional evidence is being generated. Common surrogate or intermediate clinical endpoints that have been demonstrated to predict clinical benefit include response rate, duration of response and disease-free survival. In oncology, AA is a commonly utilized approval pathway for drug evaluation for products that are determined to provide a meaningful therapeutic benefit over existing treatments [1]. AA requires that the post-marketing confirmatory clinical trials are conducted to verify benefit. In the event that the drug does not have the anticipated improvement over standard options, approval may be revoked.

In the USA, once a drug has received regulatory approval, the funding of the therapy involves patient pay, private health insurance and government programs. Individuals may be covered by plans offered by their workplace or purchase coverage independently with varying levels of reimbursement. The government plans include Medicaid and Medicare, which focus on low-income, disabled and elderly populations [2]. Guidelines for the consideration of oncology drug funding are provided by the National Comprehensive Cancer Network, although access depends on the level of health care insurance of an individual [3].

For drug approvals in Canada, a similar regulatory pathway exists within Health Canada, allowing for the issuance of a Notice of Compliance with Conditions (NOC/c) [4]. The NOC/c enables early conditional market approval for promising new drug therapies for serious, life-threatening or severely debilitating diseases, conditions for which there is no existing therapy available on the Canadian market and conditions for which there is a significant increase in efficacy or decrease in risk. Health Canada regulatory approval does not guarantee reimbursement on Canadian formularies.

Since 2011, a separate national health technology assessment (HTA) has taken place through the Canadian Agency for Drugs and Technology in Health’s (CADTH) pan-Canadian Oncology Drug Review (pCODR) [5]. The pCODR’s deliberative framework incorporates the assessment of the overall clinical benefit, the alignment with patient values and the cost-effectiveness and feasibility of adoption in the health care system. While a positive recommendation is not legally binding, pCODR recommendations allow jurisdictions to evaluate and judge whether to provide funded access to treatment in Canada.

Canada operates on a universal health care system, with each province and territory having their own health insurance plan. Under the Canada Health Act, all medically necessary health care services (hospital and doctor) must be provided by the provincial and territorial governments [6]. Cancer therapies that are approved by Health Canada and reviewed and recommended by pCODR are eligible for provincial funding. With a single-payer system, drug pricing is negotiated at the national level after a positive pCODR recommendation before moving to formulary listing in the provinces. Due to this procedure, access to therapy is feasibly simultaneous across the country.

In recent years, drug development has progressed at a rapid pace in oncology. There have been a number of new therapeutic options that have moved quickly from clinical trials to conditional regulatory approval based on promising results. AA was selected as a benchmark for the approval process as many pharmaceutical companies choose to submit regulatory files in the USA first. This study examines oncology indications authorized under an AA to Health Canada and pCODR approvals by assessing timelines and Canadian funding decisions. The goal is to evaluate the FDA’s AA indications as a point of reference for Canadian access to new drug treatments.

## 2. Methods

FDA databases were searched to identify AA malignant hematology and oncology approvals from 1 January 2000–31 December 2019. The database search was supplemented by the publication by Beaver et al. on the AA of drugs and biologics for cancer indications [7]. Health Canada databases were searched to identify if a parallel application was submitted by the sponsor, the corresponding date of the submission and Health Canada’s decision date and approval status. The date the drug met the requirements for NOC/c or Notice of Compliance (no conditions) (NOC) approval was collected.

Health technology assessments by pCODR of the AA malignant hematology and oncology indications were reviewed from the inception of the formalized national process in July 2011 to September 2020. The date of the sponsor submission, and the date and content of the final pCODR recommendation was collected. The date of the first provincial formulary listing was used as a correlate for the timing of patient access to therapy.

Administrative documents from Health Canada regarding NOC/c procedures and pCODR regarding submission guidelines and procedures were reviewed. Significant revisions and changes in process were noted to explore the impact they may have had on regulatory approval and HTA decisions.

## 3. Results

**Figure 1:**
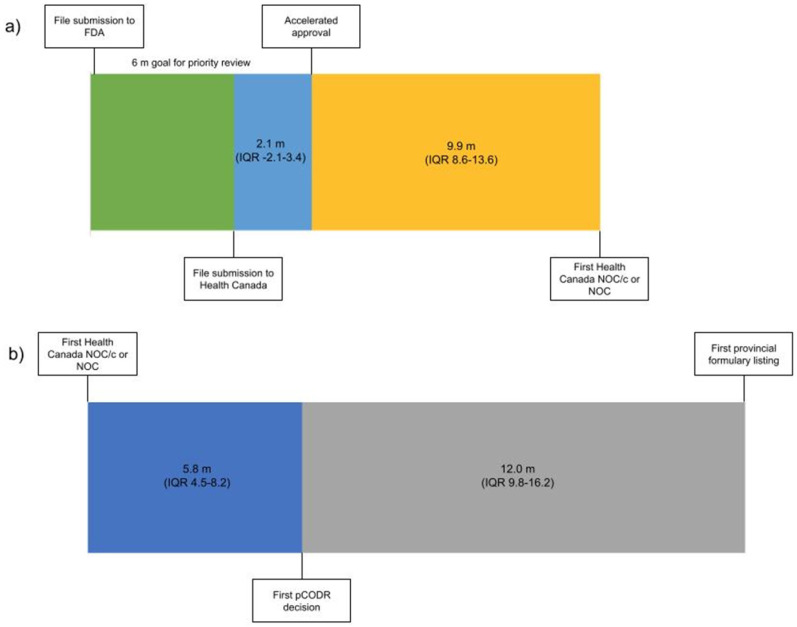
Timeline from (**a**) file submission to FDA to Health Canada NOC/c or NOC Figure 2000 (*n* = 70) and (**b**) from Health Canada NOC/c or NOC to the first provincial formulary listing after the adoption of the pCODR process 2011–2019 (*n* = 32). Median duration in months. (m—months, IQR—interquartile range).

From 1 January 2000 to 31 December 2019 there were 94 malignant hematology and oncology FDA approvals through the AA pathway, two of which were subsequently withdrawn. Of the 92 approvals, 53 received NOC/c (30 were later converted to full NOC) and 17 received NOC as their first approval status in Canada (Table S1). The 22 remaining indications have not yet been filed with Health Canada by the sponsor (Figure 1). The median time from AA to submission to Health Canada was 2.1 months preceding the AA (interquartile range (IQR) 3.4 m before to 2.1 m after AA). The median time from AA to the first NOC/c or NOC approval was 9.9 months (IQR 8.6–13.6) (Figure 1). It is of note that this time included a second review for 14 submissions following the issuance of a Notice of Deficiency or Notice of Non-Compliance by Health Canada, which would have introduced additional time into the decision based on the pharmaceutical industry’s development of a response to these notices. Of the 22 files that were not submitted for review to Health Canada, eight were for drugs with no approval in Canada and 14 were for drugs that were approved for other indications.

Health technology assessment submissions and recommendations made by pCODR were collated from 2011 to 2019 (Table S1). There were 43 NOC/c or NOC approvals during that time period; on first assessment 18 were recommended conditionally based on cost-effectiveness by pCODR, 8 were not recommended, 3 advised to re-submit, 2 were withdrawn and 12 were not submitted for HTA deliberation. Of the eight not recommended, three were subsequently resubmitted for a total of six re-submissions that were all conditionally recommended based on cost-effectiveness. The median time from the first NOC/c or NOC to the first pCODR recommendation was 5.8 months (IQR 4.5–8.2) (Figure 1). Of the 12 Health Canada approved drugs that were not submitted to pCODR, 6 were new drugs and 6 were approved drugs with a new indication. The median time from the pCODR recommendation to the first listing on a provincial formulary was 12.0 months (IQR 9.8–16.2) (Figure 1). The median time from AA to the first formulary listing for drugs that were reviewed through pCODR was 34.0 months (IQR 24.9–38.5).

**Figure 2:**
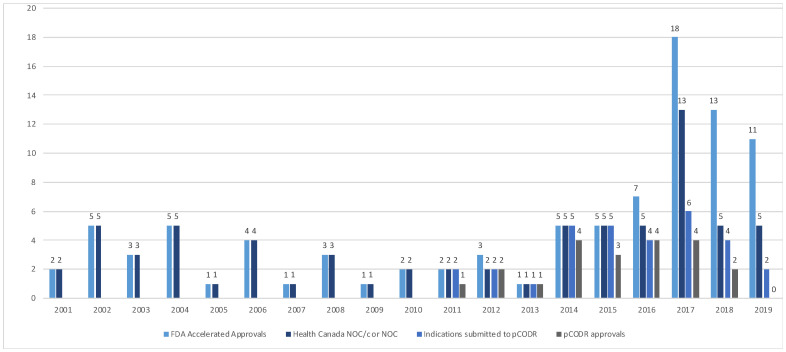
Accelerated approvals, notice of compliance issued and pCODR approvals over time from 2001–2019. (FDA—Food and Drug Administration, NOC/c—notice of compliance conditional, NOC—notice of compliance, pCODR—pan-Canadian Oncology Drug Review).

The Health Canada and pCODR approvals were examined over time (Figure 2). From 2000–2015, the FDA’s AA and HC NOC/c or NOC approvals were consistent. In 2016, a divergence between AA and sponsor submission for market authorization of new drugs or new drug indications in Canada was noted; all submissions to Health Canada of drugs that received an AA received NOC/c or NOC following review. Since the adoption of the pCODR process in 2011, there has been increasing discordance in the number of files submitted for HTA from 2016 to 2019.

**Figure 3:**
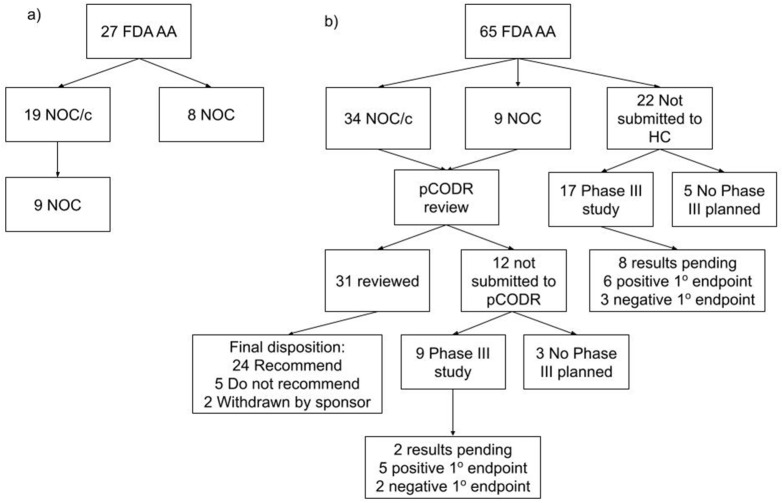
Consort diagram. (**a**) Accelerated approvals by the FDA and the corresponding Health Canada decisions from 2000 to 2010. (**b**) Accelerated approvals by the FDA and the corresponding Health Canada and pCODR decisions from 2011 to 2019. (AA—accelerated approval, FDA—Food and Drug Administration, HC—Health Canada, NOC/c—notice of compliance conditional, NOC—notice of compliance, pCODR—pan-Canadian Oncology Drug Review).

The 22 files not submitted to HC and the 12 files not submitted for HTA were examined (Figure 3). The rationale for the pharmaceutical companies’ decision not to file to either HC or HTA was not available. Of the 22 not submitted to HC, a phase III study was planned for 17 of the 22 drug indications with a primary endpoint of PFS in 10, OS in 5, co-primary endpoints of PFS and OS in 2. By the time of this study’s completion in September 2021, 8 studies were pending results, 6 met their primary endpoint and 3 did not meet the primary endpoint. Of the 12 that were not submitted to pCODR, a phase III study was planned for 9 of the 12 drug indications with a primary endpoint of PFS in 3, OS in 3, co-primary endpoints of PFS and OS in 2 and major molecular response at 12 m in one. At this study’s completion, two studies were pending results, five met their primary endpoint and two did not meet the primary endpoint.

A review of the Health Canada guidance document for NOC/c showed that it was adopted in 1998 and revised in November 2002 and September 2016. The revision in 2016 was reviewed in detail because it corresponded with the observed changes in Health Canada’s submission rate (an increase in submissions in 2017). The revision set minimum requirements for abbreviated new drug submissions for generic drugs. For all new drug submissions, revisions to annual progress reports for confirmatory trials, adverse drug reaction reporting and changes to the labelling and the marketing and educational material sections were included. The 2016 changes to the Health Canada NOC/c program were not anticipated to alter regulatory decision making.

The pCODR procedures and guidance document was published in May 2011. Multiple revisions were undertaken annually from 2016 to 2019. In 2017, as a change in the Health Canada and pCODR submission rate occurred when compared with the number of applications in the USA, the procedure and submission guideline revisions for 2016 were reviewed in detail. There were four key changes in the pCODR procedures: (1) the mandatory disclosure of submitted drug price was introduced, (2) the adoption of a common CADTH recommendation framework for both oncology and non-oncology drugs with three recommendations: reimburse, reimburse with clinical criteria and/or conditions or do not reimburse, (3) a minimum period of 120 calendar days for advance notification of anticipated submissions and resubmissions was established and (4) formal pCODR engagement with the pan-Canadian Pharmaceutical Alliance (pCPA), responsible for price negotiations, was initiated.

## 4. Discussion

A review of 20 years of AA in the USA showed that Canadian regulatory approvals aligned with the FDA decisions from 2000 to 2015. Thereafter, there was an increasing discordance in the number of AA and pharmaceutical company submissions to Health Canada. Similar trends were noted with Health Canada approvals and pCODR submissions. The combination of the pharmaceutical companies’ decision not to submit to Health Canada and/or pCODR and the more complex Canadian pharmaceutical access landscape may have led to the disparity in the access to promising oncology therapies when compared with the FDA’s AA. However, once a new drug has received regulatory approval and a positive HTA recommendation with a national price established, the treatment becomes available to the majority of Canadians through the universal health care program.

The FDA offers a number of programs to facilitate and expedite the development and review of new drugs including fast-track designation, breakthrough therapy designation, accelerated approval and priority review designation [1]. These programs focus on speeding up access to promising new therapies while balancing standards for safety and effectiveness. For the purpose of this study, accelerated approval was selected for comparison with the Canadian system based on oncology approvals through this mechanism and the similarities to NOC/c. This pathway has existed in some form since 1992 in the USA and was a model for early approvals of promising therapies. Our analysis noted a divergence between AA and Health Canada and/or pCODR submissions in more recent years, predominantly due to sponsors choosing not to submit for review. A review of the guidance documents for both Health Canada and pCODR suggests that they are not expected to have contributed to the difference. Evolution in other pharmaceutical industries’ global submission planning approach and the development of real-world evidence standards may also have impacted the decision to submit.

In Canada’s publicly funded healthcare systems, HTA aims to optimize the balance of health outcomes and the allocation of scarce resources with increasingly strained budgets. The process allows a systematic evaluation of the clinical effectiveness, safety, patient value, and cost-effectiveness of a new therapy to inform decision making. Single-arm studies are often the basis of AA approval, and conducting an HTA review with these data is challenging and suboptimal. This difficulty is noted across the HTA countries and efforts to adapt evaluation include the incorporation of real-world evidence and modified processes [8]. For example, in the UK, the National Institute for Health and Care Excellence (NICE) has developed a separate procedure for Orphan Medicinal Products (OMPs). The Highly Specialised Technology program acknowledges the complexity of assessing value with non-randomized controlled trials, amongst other limitations, and has adopted a modified approach to standard HTA approaches [9]. In Canada, an alternate program for these situations has not yet been developed. Pharmaceutical companies may recognize that the level of uncertainty around clinical benefits in the absence of trial comparator data may result in a Canadian HTA recommendation not to reimburse. This may account in part for the decision of sponsors to not bring their product early to Canada.

The review times within regulatory and HTA bodies, respectively, in Canada were within their prescribed standards. From file submission to decision, the Health Canada process took a median of 10.2 months. The Health Canada standards are 180 days for a priority review, 200 days for therapies that have been granted advanced consideration for NOC/c and 300 days for a standard review. This aligns with the FDA timelines, where the Prescription Drug User Fee Act outlines a 10-month standard review process goal and 6 months for a priority review [10]. However, the time to reach a regulatory decision in Canada generally lags behind the USA, as the applications are submitted in the USA before Canada. The introduction of initiatives such as Project Orbis, whereby multiple regulatory agencies collaboratively review applications, will address some of the timeline concerns for approvals for oncology therapies [11].

From 2011 forward, when HTA was formally incorporated into the decision-making process, regulatory approval to a final pCODR decision had a median of 5.8 months. To shorten timelines, pCODR adopted an option that permitted file submission before NOC/c or NOC, provided that Health Canada approval was anticipated within 6 months, fitting with the aligned reviews between Health Canada and health technology assessment organizations [12].

The longest delay was from pCODR decision to formulary listing, due in part to pCPA price negotiations. In 2018, to address this issue, pCPA set target timelines for deliverables, including the delivery of an engagement letter within 10 days of HTA recommendation, pricing consideration within 40 days of HTA recommendation and from negotiation to the letter of intent within 90 days of issuing the engagement letter [13]. With this revised timetable, pCPA met the targets in over 90% of submissions in 2019. Multiple procedural changes have been implemented to assist in reducing delays for new treatment options for Canadians, thereby improving social welfare [14].

Evaluating the economic, patient and system impacts of a new treatment through HTA may introduce more delays, but it is a necessary approach for sustainability in a universal health care system [15,16]. In our review, 48 of the 65 AAs from 2011 to 2019, after HTA implementation in Canada, had phase III confirmatory studies completed or in progress. While 30 indications were verified and 10 had results pending at the time of this study’s conclusion, 8 did not achieve the designated primary endpoint. If these latter programs had been implemented, the process of disinvestment would have been a significant undertaking.

Challenges with re-evaluation and disinvestment are demonstrated by “dangling” AA in oncology, whereby the required clinical trials did not end up confirming any benefit, but the marketing authorization continued [17]. Ten immune checkpoint inhibitor indications fell under this dangling categorization, for which the sponsors voluntarily withdrew four; however, six were voted on by the Oncology Drugs Advisory Committee and four were recommended to remain on the market. Interestingly, for the 10 dangling AAs, 5 were not submitted to Health Canada. The remaining five received Canadian regulatory approval; however, none of these products received a positive HTA recommendation. The decision in the USA to allow these therapies for an indication that did not confirm a benefit has resulted in patients continuing to receive treatment, with financial investment from their health care insurance, that may not improve their outcomes. These decisions, in addition to other controversial recommendations such as aducanumab for Alzheimer’s disease, have raised questions about the AA program [18]. With aducanumab, the FDA approved the AA indication despite the negative feedback from the expert advisory committee, which may have wider implications in oncology [19]. With new drug development, there is an increasing tension between early access and adequate evidence at the regulatory level. Powell et al. note that enforcing and strengthening the FDA post-marketing requirements would support the generation of quality data that confirm benefit for patients [20]. While HTA may be seen as a barrier to access, the counterpoint must be acknowledged in that it provides an in-depth evaluation that limits recommendation if the level of uncertainty is high.

Resolving uncertainty around clinical benefit through randomized trials is ideal; however, it is challenging for smaller populations where phase III trials are not feasible, for example genomic-based therapies. Additional impediments include the rapid adoption in clinical practice of new treatments in some countries, resulting in the inability to generate the type and quality of data needed for HTA. These scenarios pose difficulties for the Canadian landscape and require innovation in methodology to try to address the gap. Real-world evidence methods and access to evidence-development programs may allow patients to receive promising new drug treatments while generating the evidence to enable a decision to disinvest or invest in therapy.

## 5. Conclusions

Our study highlights the challenges that Canadian clinicians and patients face in terms of timely access to promising new cancer therapies granted an FDA AA. Non-simultaneous regulatory submissions followed by health technology assessment for reimbursement results in funded access almost 3 years after AA. Access in Canada, however, is associated with greater certainty of clinical benefit and cost effectiveness and incorporates a consideration of health care system sustainability. With the added layer of HTA, pharmaceutical companies may be deterred from bringing a product to Canada due to concerns about a negative recommendation for funding; however, this may be an unavoidable penalty for providing funded health care to all Canadians. Domestic and international collaboration amongst regulators and downstream healthcare partners will help to decrease time delays between US regulatory approvals and Canadian patient access.

## Data Availability

The data presented in this study are available on request from the corresponding author.

## Supplementary Materials

The following supporting information can be downloaded at: https://www.mdpi.com/article/10.3390/curroncol29020036/s1, Table S1: AAs for Malignant Hematology and Oncology Products and Canadian Regulatory and Health Technology Assessment decisions and timelines from 2001 to 2019. (FDA—Food and Drug Administration, AA—Accelerated approval, RR—response rate, DFS—disease free survival, MRD—minimal residual disease, DOR—duration of response, CLL—chronic lymphocytic leukemia, CML—chronic myelogeous leukemia, BC—blast crisis, CP—chronic phase, AP—accelerated phase, NHL—non Hodgkins lymphoma, HR—hormone receptor, Ph—Philadelphia, NSCLC—non small cell lung cancer, MM—multiple myeloma, SCT—stem cell transplant, EGFR—epidermal growth factor receptor, mCRC—metastatic colorectal cancer, RCC—renal cell carcinoma, GIST—gastrointestinal stromal tumor, HL—Hodgkins lymphoma, ASCT—autologous stem cell transplant, ALK—anaplastic lymphoma kinase, TKI—tyrosine kinase inhibitor, ALL—acute lymphocytic leukemia, BRAF—B-Raf proto-oncogene serine/threonine kinase, HER2—human epidermal growth factor receptor 2, PDL1—Programmed death-ligand 1, HNSCC—head and neck squamous cell carcinoma, STS—Soft-tissue sarcomas, BRCA—BReast CAncer gene, 5 FU—5 fluorouracil, CPS—combined positive score, MSI-H—microsatellite instability-high, DMMR—mismatch repair deficient, HCC—hepatocellular carcinoma, AML—acute myeloid leukemia, NTRK—Neurotrophic tyrosine receptor kinase, DLBCL NOS—diffuse large B cell lymphoma not otherwise specified, SCLC—small cell lung cancer, MCL—mantle cell lymphoma, NOC(/c)—Notice of Compliance (/conditional), pCODR—pan Canadian Oncology Drug Review, HC—Health Canada).
